# An atomistic view of Hsp70 allosteric crosstalk: from the nucleotide to the substrate binding domain and back

**DOI:** 10.1038/srep23474

**Published:** 2016-03-30

**Authors:** Federica Chiappori, Ivan Merelli, Luciano Milanesi, Giorgio Colombo, Giulia Morra

**Affiliations:** 1 Istituto di Tecnologie Biomediche – CNR, Segrate (Mi), 20090 Italy; 2 Istituto di Chimica del Riconoscimento Molecolare – CNR, Milano, 20131 Italy

## Abstract

The Hsp70 is an allosterically regulated family of molecular chaperones. They consist of two structural domains, NBD and SBD, connected by a flexible linker. ATP hydrolysis at the NBD modulates substrate recognition at the SBD, while peptide binding at the SBD enhances ATP hydrolysis. In this study we apply Molecular Dynamics (MD) to elucidate the molecular determinants underlying the allosteric communication from the NBD to the SBD and back. We observe that local structural and dynamical modulation can be coupled to large-scale rearrangements, and that different combinations of ligands at NBD and SBD differently affect the SBD domain mobility. Substituting ADP with ATP in the NBD induces specific structural changes involving the linker and the two NBD lobes. Also, a SBD-bound peptide drives the linker docking by increasing the local dynamical coordination of its C-terminal end: a partially docked DnaK structure is achieved by combining ATP in the NBD and peptide in the SBD. We propose that the MD-based analysis of the inter domain dynamics and structure modulation could be used as a tool to computationally predict the allosteric behaviour and functional response of Hsp70 upon introducing mutations or binding small molecules, with potential applications for drug discovery.

The Hsp70 chaperone family plays a central role in mediating correct protein folding as well as buffering the cell toxicity of denatured and misfolded polypeptides^1^. Human cells possess cytosolic members that are constitutively expressed and have housekeeping functions, such as Hsc70, organelle-specific Hsp70, such as GRP75 and GRP78, and stress-induced Hsp70. The latter, together with other members of the Hsp family (including Hsp90, Hsp40 and Hsp27), mediates the stress response: it participates in the heat shock pathway that is activated to restore normal protein folding under stress conditions, thus preventing cell death, and is therefore an essential player in cell homeostasis^1^,^2^. Dysregulation of Hsp70-related pathways is implicated in a number of diseases, including neurodegenerative disorders and cancer^3^. In particular, the strong dependence of cancer cells on the heat shock pathways and the correlation between overexpression of Hsp70 and poor prognosis has spurred significant efforts in targeting this system with potential drugs^4^,^5^. In this scenario, as well as for its relevance in neurodegenerative conditions^6^,^7^, Hsp70 emerges as a major potential drug target for multiple diseases. To this end, understanding the functional mechanisms and details of its allosteric behaviour holds great promise for advancing drug design approaches and expanding therapeutic opportunities.

From the structural viewpoint Hsp70s are highly conserved proteins comprising two domains, connected by a flexible linker: the N-terminal nucleotide binding domain (NBD) with ATPase activity, and the substrate binding domain (SBD), which is made of one β subunit (βSBD), hosting the substrate binding site, and one α subunit (αSBD) forming a “lid” (Fig. 1).

The allosteric modulation that governs the Hsp70 folding function^8^ relies on a bidirectional communication between SBD and NBD regulating the conformational cycle^9^,^10^,^11^,^12^ (Fig. 1). In detail, the ADP/ATP switch in the NBD stimulates in the SBD the transition from the high affinity closed-lid to the low affinity open-lid state, favouring the release of substrate. In the opposite direction, binding and release of substrates, typically linear hydrophobic polypeptides or exposed regions of partially folded proteins^13^, has been shown to induce the transition to the ATP state, stimulating the ATPase^14^,^15^,^16^. *In vivo*, the cycle is assisted by various co-chaperones such as nucleotide exchange factors (NEF), which help replacing ADP with ATP^17^,^18^,^19^, and J proteins (Hsp40s) that present the substrate to the SBD^19^. The latter modulate the conformational cycle resulting in enhanced ATPase, possibly by stabilizing an intermediate, as observed *in vitro* in the presence of ATP and of the substrate peptide NR in ref^11^.

Hsp70 is highly conserved among different species, with the human Hsp70 and the bacterial DnaK sharing a sequence identity of about 50%. DnaK constitutes a valuable model system to illuminate the conformational mechanisms underlying the chaperone function and its allosteric properties since high resolution atomic structures have been published for both equilibrium endpoints of the conformational cycle, namely the ADP-bound (closed)^20^ and the ATP-bound (open) conformation^14^,^21^ (Fig. 1).

Although the communication from the NBD to the SBD has been elucidated in some detail^9^,^12^,^22^,^23^,^24^, highlighting relevant residues and conformational changes inducing the ADP to ATP state transition, the allosteric mechanism in the opposite direction has been less explored so far. Recent data have provided insight into the conformational response of the SBD upon substrate binding, and have connected it to its allosteric behavior^24^, but a comprehensive mechanistic model is still lacking. In particular, it is not clear yet how the conformational response of DnaK can change depending on the specific combination of NBD-bound and SBD-bound ligands, and ultimately how, at the molecular level, the signal encoded by peptide-substrate binding is propagated from the SBD to the NBD and facilitates inter domain docking. The availability of high-resolution crystal structures allows us to address these two questions by using all atom Molecular Dynamics (MD) simulations.

We focus on the analysis of the conformational and dynamical modulation induced in the ADP-bound, high affinity structure upon exchanging the nucleotide from ADP to ATP and at the same time considering different ligand states in the SBD. By comparing the conformational evolution of the different MD trajectories, we are able to highlight the conformational and dynamical modulation locally induced by a specific combination of ligands. Relying on these simulations, we propose a model of the interdomain communication underlying the experimentally observed conformational changes. According to it, the NBD-bound nucleotide affects the linker orientation through a set of conformational rearrangements involving lobe II, in agreement with experimental data^22^ and with our previous study^25^. On the other hand, the SBD bound peptide locally modulates the conformational rigidity of the C-terminal end of the linker. In the presence of ATP this facilitates the alignment of the beta SBD, favouring the linker docking, possibly related to the enhancement of ATPase.

## Results and Discussion

The high affinity, closed DnaK structure (PDB code: 2KHO^20^), representing the ADP bound state, was used as starting conformation and simulated in 6 different ligand states, resulting from having ADP or ATP in the nucleotide binding site in combination with one of three different states of the SBD, namely apo, bound to the substrate peptide NRLLLTG (NR)^26^, and bound to the inhibitor peptide RPVYIPRP (Api88)^27^. Each system is referred in the following as NBD(ADP/ATP)-SBD(free/NR/Api88) to indicate the NBD and the SBD ligand states, respectively.

**Figure 2:**
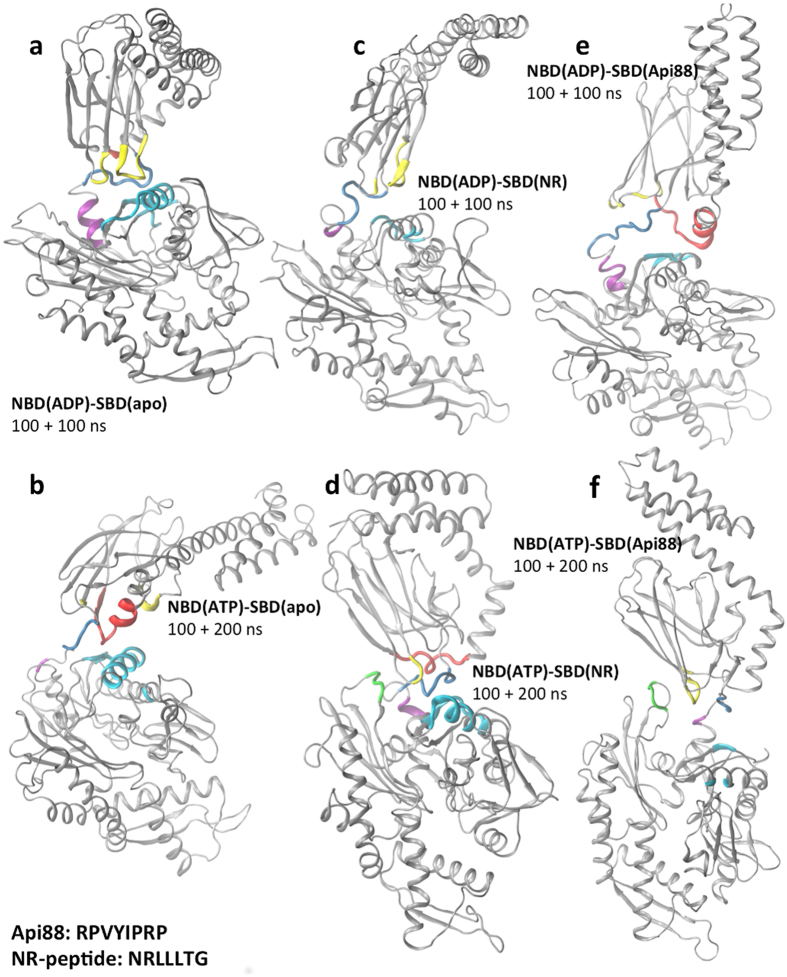
Contact interface. Representative conformation of the most populated cluster for each complex is displayed: (**a**) NBD(ADP)-SBD(free); (**b**) NBD(ATP)-SBD(free); (**c**) NBD(ADP)-SBD(NR); (**d**) NBD(ATP)-SBD(NR); (**e**) NBD(ADP)-SBD(Api88); (**f**) NBD(ATP)-SBD(Api88). Regions that correspond to the contacting interfaces between NBD and SBD along the trajectories (see Methods) shown in the colour of the subdomain they belong to (see legend Fig. 1).

For each complex, two independent trajectories were produced, with a total simulation time of 200 ns for the ADP complexes and 300 ns for the ATP complexes, reaching 1.5 microseconds for the whole dataset (Fig. 2). The aim of our simulations is to investigate the role of the substrate/inhibitor in inducing a modulation of the DnaK conformation, when combined with a given nucleotide in the NDB site. We can consider the NBD(ADP)-SBD(free) system as a reference state, populating at equilibrium an ensemble of conformations similar to the starting crystal structure, and investigate the response induced by switching nucleotide or ligand.

### Interdomain arrangement

#### Nucleotide-bound systems

We first focus on two systems: NBD(ADP)-SBD(free), and NBD(ATP)-SBD(free), where ADP is replaced by ATP. In Fig. 2a,b we show the representative structure of the most populated conformational cluster of each system. The SBD maintains a fairly conserved orientation relative to the NBD in all the ADP trajectories (see Fig. 2a and S1a). In contrast, the replacement of ADP with ATP induces a reorientation of the SBD with respect to the NBD, resulting in a higher conformational freedom (see Fig. 2b and S1b). In fact, multiple replicas of the same system evolve towards different directions for the orientation of the SBD domain relative to the NBD. The different relative orientation of the SBD with respect to the NBD in the two nucleotide states is reflected by a different composition of the contact interface. In the NBD(ADP)-SBD(free) complex, from the SBD side, loops of the βSBD are involved in contacts with the NBD, whereas with ATP also the HA helix of the αSBD takes part in defining interdomain contacts (Table S1). From the NBD side, in the ADP case, the βSBD contacts the NBD subdomain IIB (residues 225–310) in all the trajectories, whereas when ATP is bound to the NBD, an involvement of subdomain IIA in the NBD-SBD interface emerges (Table S1).

**Figure 3:**
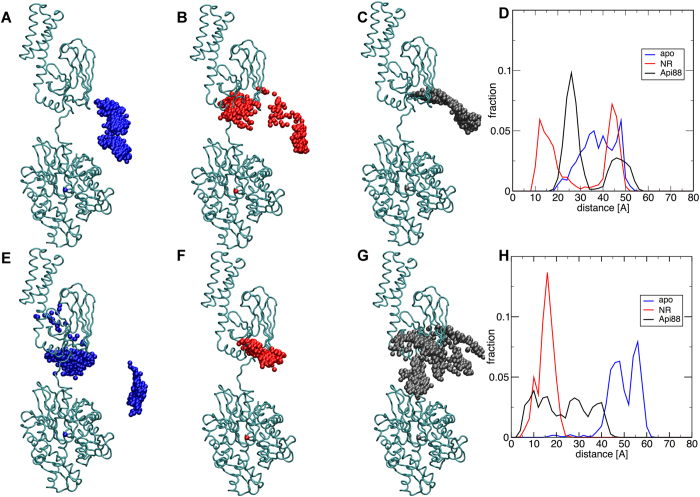
Distribution of the SBD centre of mass along the MD trajectories, after aligning the protein to the NBD. Top, cloud showing the distribution of SBD centre of mass coordinates relative to the starting conformation for ADP bound systems: (**A**) SBD(free), (**B**) SBD(NR), (**C**) SBD(Api88). Bottom, same for ATP bound systems: (**E**) SBD(free), (**F**) SBD(NR), (**G**) SBD(Api88). (**D**) Distribution of distances from the starting position of the SBD centre of mass for ADP bound systems, obtained after merging the two independent runs for each system (blue: SBD(free), red SBD(NR), black SBD(Api88). (**H**) Same for ATP bound systems.

In order to quantitatively characterize the difference between the two nucleotide states, we calculated the time evolution of the SBD centre of mass along the MD trajectories. As expected, the results highlight a complex distribution for ATP, with larger width and at least two ensembles (see Fig. 3A,B and Supplementary Fig. S1). The distribution of SBD centre of mass coordinates can be characterised by their distance from the starting position: this quantity measures the dynamic flexibility of the interdomain arrangement. The average distance goes from 37 Å in the case of ADP to 49 Å in the case of ATP (Fig. 3G,H). The associated pair distance distribution of the ensemble (Supplementary Fig. S2), has a peak at 14 Å for ADP and 46 Å for ATP. Both parameters suggest that the presence of ATP reorients the SBD position more strongly than ADP.

#### Nucleotide and substrate peptide-bound ternary complexes

In the presence of the nucleotide in the NBD, the substrate peptide NRLLLTG was inserted into the SBD in the starting structure 2KHO, and ternary complexes NBD(ATP/ADP)-SBD(NR) were simulated under the same conditions as reported above. The representative conformations of the most populated clusters are shown in Fig. 2c,d. In the presence of ADP, the NR-peptide does not affect the contact surface in the representative conformation, while in the ATP complex the NR-peptide bound to the SBD results in the involvement of the loop210 (res. 209–214) in the contact surface, as compared to the NBD(ATP)-SBD(free) complex, where this arrangement was observed only in one trajectory, thereby amounting to about 30% of the simulation time. Compared to the peptide free complexes, here the SBD position relative to the NBD is differentially modulated, as shown by the centre of mass distributions (Fig. 3 and Supplementary Fig. S1,S2). More in detail, in the presence of ADP (Fig. 3C,G), we observe that the distribution of SBD centre of mass positions of the NBD(ADP)-SBD(NR) significantly overlaps to that of the NBD(ADP)-SBD(free) system (average distance from the starting vertical position: 28 Å in NBD(ADP)-SBD(NR), 37 Å in NBD(ADP)-SBD(free)), although in the pair distance distribution peaks are at 41 and 27 Å with and without peptide, respectively (Supplementary Fig. S2). It is worth mentioning that the bimodal shape of the NBD(ADP)-SBD(NR) average distance distribution reflects the presence of MD replicas evolving in different directions after equilibration, suggesting a highly flexible system. In the presence of ATP, in contrast, the collapse of the SBD over the NBD, observed in the NBD(ATP)-SBD(free) and resulting in a wide distribution of centre of mass positions, does not occur in the NBD(ATP)-SBD(NR) case. Here the SBD spans a limited range of orientations, located over the top of the NBD, in a parallel, face-to-face arrangement of the two DnaK domains (Fig. 3D,G). The average distance from the starting structure (14 Å, Fig. 3G) and the peak pair distance 11 Å (Supplementary Fig. S2), not only are narrower here than in the NBD(ATP)-SBD(free) case, but also relative to both ADP-bound systems. This suggests that the combination of peptide substrate and ATP specifically reduces the relative conformational freedom of the two domains.

#### Nucleotide and inhibitor bound ternary complexes

An equal number of trajectories were run in the presence of the inhibitor peptide, Api88, bound to the SBD, using the same protocol as for the above reported systems. Here, similar to the case of substrate peptide, the involvement of loop210 in the domain contact interface is observed in the ATP complex (Fig. 2e,f). Also, the prevalence of the face-to-face interdomain arrangement in both nucleotide states is observed in the representative structures of the most populated cluster (Fig. 2e,f) as well as in the centre of mass distribution (Fig. 3E,F, S2). However, a difference is found in the interdomain arrangement of NBD(ATP)-SBD(NR) and NBD(ATP)-SBD(Api88). The latter spans a wider region than the substrate bound case (average distance: 22 Å and pair distance peak 16 Å), suggesting an intermediate behaviour between the SBD(free) and the SBD(NR) case. In contrast, the distributions of centre of mass observed in the presence of ADP are more similar for all SBD ligand states in terms of average distance from the starting point (28 Å and 32 Å for NR and Api88, respectively, see Fig. 3G,H).

Overall, we observe that ATP induces a strong modulation of the SBD positioning, depending on the presence or absence of the peptide in the SBD binding site. In particular, without the peptide, the SBD collapses over the NBD, whereas in the presence of the SBD ligand the two domains are more rigidly aligned. In contrast, in NBD(ADP)-SBD(free/ligand) systems no significant differences are observed depending on the SBD ligand state. This points to the nucleotide as an essential factor activating the allosteric interplay between NBD and SBD.

### Linker conformations and contacts

The previously discussed interdomain arrangements may ultimately depend on the conformational dynamics of the linker (residues 383–396) connecting the terminal helix of the NBD to the βSBD. Based on this assumption, here we investigated how the structural population of the linker region is locally affected by each ligand.

At the single residue level, Tables 1 and 2 show the contacts and the hydrogen bonds involving the linker and persistent during each MD trajectory. As for the NBD residues, the linker stably contacts the terminal strand of lobe IA (residues 165–170) and the NBD-terminal helix (residues 378–382). The SBD amino acids involved in the interaction include loop L_6,7_ (residues 479–483, Asp481) and loop L_2,3_ (residues 412–420), as well as residues around 500–510 of the αSBD. Other sets of contacts are present only in specific ligand states. When comparing ATP- to ADP-bound systems, either in the presence or absence of ligands at the SBD, the most evident nucleotide dependent factor is the involvement of loop210 of the NBD: ATP favours interactions of linker residues with loop 210, but these interactions are not found with ADP. This confirms our previous results^25^ highlighting the allosteric effect of ATP in stabilizing a conformation of loop210 that orients towards the linker and sustains its interaction with the NBD.

Next, we focused on the ligand-induced structural modulation of the linker. In order to do that, a clustering procedure was applied to a meta-trajectory, obtained by concatenating all MD runs regardless of their ligand state, but considering only the residue interval (373–433) This stretch of residues includes the terminal helix of the NBD, the linker and the three first strands of the SBD, including the beta hairpin L_1,2_ (residues 404–406) that contacts the ligand in the binding site. The aim of this analysis is first to identify the most populated common linker structures visited in all simulations, and then to reconnect them to single trajectories and therefore specific combinations of ligands. Using the cluster decomposition on the meta-trajectory instead of analysing each complex separately allows us to univocally connect simulations showing similar features and extrapolate the effect of each ligand or pair of ligands.

#### NBD domain/linker interface

**Figure 4:**
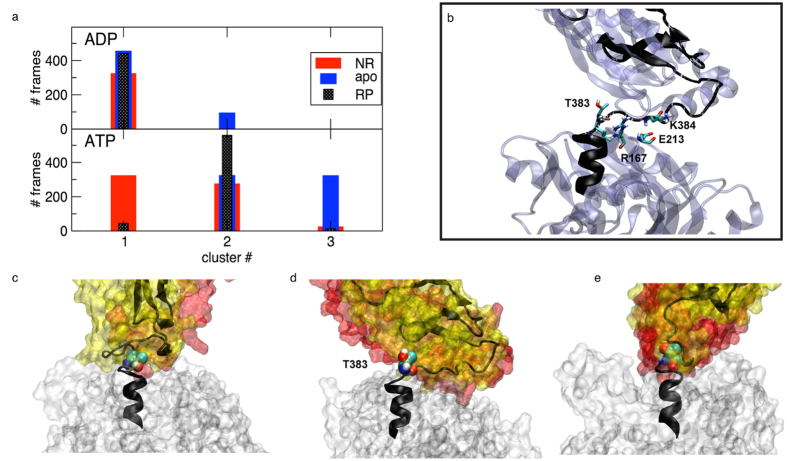
Cluster analysis of the linker (370–393). (**a**) histogram showing the population of the dominant three clusters according to the nucleotide and ligand state (Top: ADP bound systems, bottom: ATP bound systems. RP indicates the Api88-bound state). (**b**) Close-up of the representative conformation of the second cluster, showing the arrangement of relevant residues Th383, of Glu213 and Arg167. (**c**–**e**) representative conformations of the dominant three clusters (from left to right: first, second, third) showing the terminal C helix in grey cartoon, the NBD in grey surface, the beta and alpha SBD in yellow and red surface, respectively, and highlighting the arrangement of Thr383.

We first focused on the NBD + linker segment corresponding to residues 373–393, the first half of the residue stretch defined above. The population fraction of the first 3 clusters, broken down for each ligand state is shown in Fig. 4a. The conformational preference of segment 373–393 is strongly affected by the nucleotide binding state, since all ADP bound systems prevalently fall into cluster 1 (Fig. 4c), whereas the ATP bound systems mainly populate cluster 2 (Fig. 4d), with the apo form also significantly occupying cluster 3 (Fig. 4e). In contrast, the SBD ligand appears less critical for the conformational preference of this segment. More in detail, ADP-bound systems (cluster 1) mainly have an intact C-terminal helix extending to residue Thr383, which differs from the starting crystal conformation only for the bending of the linker. In contrast, all ATP bound systems consistently populate a structure in which the last turn of the C-terminal helix is unfolded. Here the backbone of Leu382 and Thr383 interact with Arg167 and this induces the psi angle of Thr383 to populate a cis conformation (average value in ATP bound systems is 103° versus 42° for the ADP systems). At the same time Lys387 interacts with residues of loop210 (see details in Fig. 4b). This explains how Arg167 and loop210, which are positioned on lobe I and lobe II respectively, respond to the nucleotide state and affect the linker.

#### Linker/SBD domain interface

**Figure 5:**
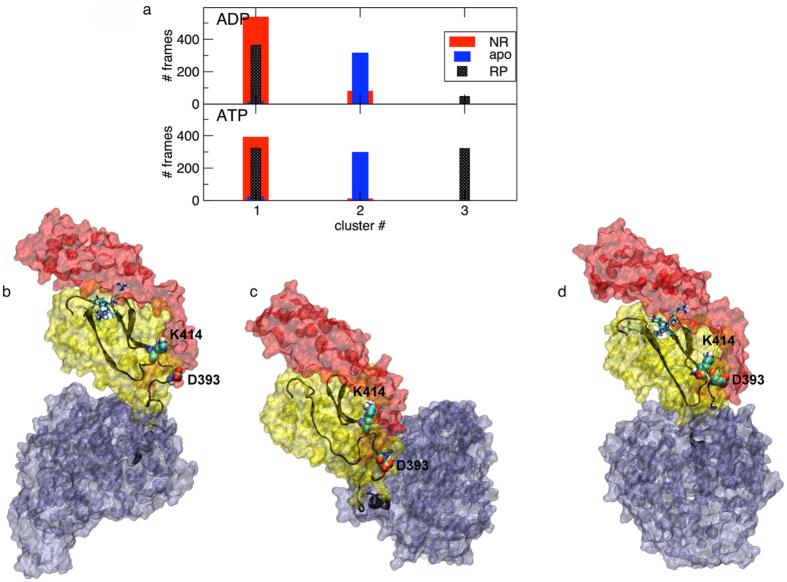
Cluster analysis of the linker-βSBD (393–413). (**a**) histogram showing the population of the dominant three clusters broken down according to the nucleotide and ligand state (Top: ADP bound systems, bottom: ATP bound systems. RP indicates the Api88-bound state). (**b**–**d**) representative conformations of the dominant three clusters (from left to right: first, second, third) highlighting the analysed region in grey cartoon, the NBD in grey surface and the beta and alpha SBD in yellow and red surface, respectively.

We repeated the clustering on the same meta-trajectory considering the residue interval 393–433. This segment includes the C-terminal end of the linker and the first strands of the SBD beta domain, reaching the substrate binding site loop L_1,2_, as well as L_2,3_ facing the NBD interface. Again, once the most populated conformers were identified through the cluster analysis, the ensembles were decomposed according to the contribution of each ligand state (Fig. 5a). Here we find that the presence of a peptide bound to the SBD induces a prevalent conformation that defines cluster 1 (Fig. 5b), whereas the absence of a ligand favours the conformation represented in cluster 2 (Fig. 5c). Moreover, cluster 3 shows an alternative conformation that is only populated in the presence of the inhibitor Api88 (see Fig. 5d). Interestingly, the presence of a specific nucleotide (ATP or ADP) in the NBD does not significantly affect the linker-SBD cluster population, suggesting that the structure of this region mainly depends on local interactions that are driven by the substrate. In detail, in the presence of a bound peptide we observe the increase of stable contacts between the linker (residues 384–396) and loop L_2,,3_ (see Table 2). We hypothesized that these contacts might be stabilized because of the enhanced internal dynamical coordination of the SBD loop L_2,3_ induced by the ligand. To test this hypothesis, we calculated the distance fluctuation map of the domain (Supplementary Fig. S3). This analysis reports on the internal rigidity within the protein by evaluating the fluctuation of pair distances for each couple of Cα atoms. We find that residues around Pro396 markedly increase their coordination relative to the adjacent loop L_2,3_ and β-strand 3 when going from the SBD unbound to the SBD bound state. This substrate-induced increased rigidity is associated with a reduced conformational freedom of the phi angle between Asp393-Val394, which stabilizes a parallel interdomain arrangement, in all nucleotide states (Supplementary Fig. S3). In this respect, substrate peptide and inhibitor generate a similar dynamical response. In contrast, the absence of SBD ligands destabilizes the interaction between L_2,3_ and linker and increases the conformational freedom of the hinge Asp393-Val394, resulting in the collapse of the SBD over the NBD, represented by cluster 2 (Fig. 5c).

Overall, the cluster analysis, performed on two residue subsets connecting NBD and SBD allows us to dissect the directions of propagation of the allosteric message from the nucleotide to the substrate binding domains and vice-versa. The inclination of the SBD relative to the NBD appears to depend strongly on the nucleotide. The cluster analysis on residues 373–393 shows that ADP leaves the linker orientation substantially unaffected in comparison to the starting structure, whereas ATP induces a twist at Thr383, through the simultaneous interaction with lobe I (Arg167) and lobe II (loop210). On the other hand, the absence of a SBD substrate maintains the conformational freedom of the C-terminal end of the linker towards the SBD, resulting in a wide distribution of centre of mass positions. In contrast, when the peptide is present, the nucleotide-induced rearrangement of the linker can be balanced by the coordinating effect of the substrate. In fact, the analysis on residues 393–433 shows that a substrate in the SBD binding site induces a partial alignment of the beta SBD to the C-terminal end of the linker, in a way that is weakly dependent on the nucleotide, at least in the early stage of docking conformations sampled by our MD trajectories. This rigidity is coupled to the parallel domain arrangement of NBD and SBD. When ATP is bound at the NBD and a substrate is present in the SBD binding site, we observe a narrower conformational space for the linker and a particularly tight global arrangement of the two DnaK subdomains. In this context, the inhibitor Api88 acts similarly to peptide NR: it increases the internal coordination in the SBD and favours the alignment between NBD and SBD, by stabilizing the conformation of residues 393–433 represented by cluster 1 (Fig. 5b). Moreover, it can induce the population of an alternative SBD structure, corresponding to cluster 3, which is reflected, in the case of the NBD(ATP)-SBD(Api88) system, in a wider distribution of centre of mass positions than in NBD(ATP)-SBD(NR) (Fig. 3).

The dramatic effect on the inter-domain arrangement caused by local modulations at Thr383 and Asp393 suggests that, in line with the theoretical framework proposed in a recent paper^28^, local fluctuations on a short time scale occurring at flexible linker regions can help overcome free energy barriers that separate globally different conformational states.

### Structural evolution of the conformation of peptide-ATP-DnaK complex

**Figure 6:**
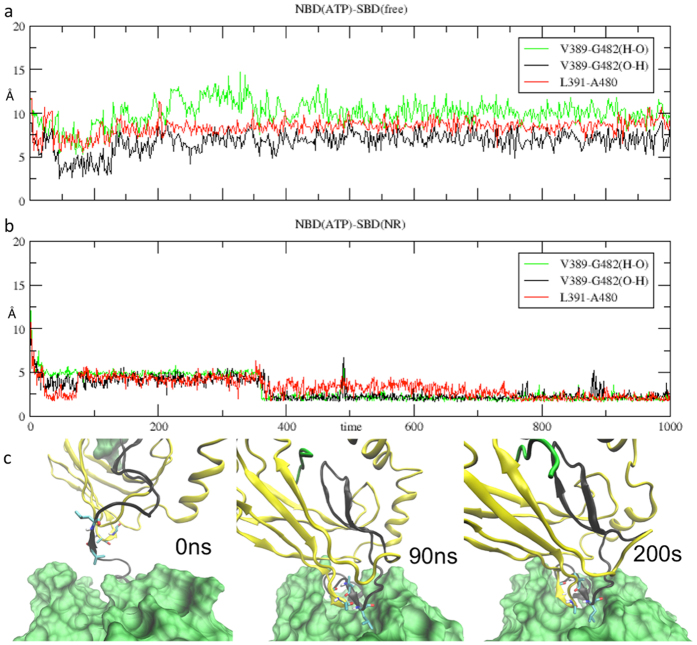
Linker docking. Plot of distances between linker and loop L_6,7_ residues in NBD(ATP)-SBD(free) complex on top, and in NBD(ATP)-SBD(NR), in the middle. Screenshots of NBD(ATP)-SBD(NR) complex at the bottom, show the short β-sheet composed by the linker and by loop L_6,7_.

When bound to the NR peptides, replicas of the ADP complexes display a significant conformational variability, which is reflected in a high RMSD value between snapshots (Supplementary Fig. S4). In contrast, complexes bound to ATP show high consistency and a relatively low RMSD between the two replicas (Supplementary Fig. S4b), due to a common orientation of the SBD domain. This allows us to define a structural ensemble for the NBD(ATP)-SBD(NR) complex, represented by the conformation in Fig. 2. An essential feature of this conformation is the presence of a short β-strand (Val398-Leu391), formed by the linker in the NBD pocket delimited by lobes IA and IIA. In particular, in the 200 ns simulation of the NBD(ATP)-SBD(NR) complex, this strand is formed at the beginning of the trajectory, and it becomes more stable as it pairs to a second strand formed within the loop L_6,7_ of the SBD, after about 70 ns of simulation (Fig. 6). It is worth noting that the β-strand content of the linker residue stretch is markedly increased in the simulations of the NBD(ATP)-SBD(NR) complexes (34%) compared to the Api88 case (21%), and also slightly higher than the SBD(free) complex (32%). The β-sheet is maintained by several H-bonds between the highly conserved^11^Val389 and Gly482 and between Leu391/Leu392 and Ala480 (Fig. 6).

**Figure 7:**
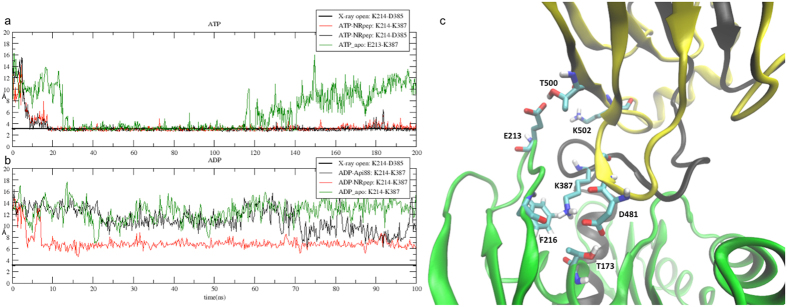
SBD docking and interactions between linker and loop210. Plot of the distance between loop210 and linker residues in ATP complexes (**a**) and in ADP complexes (**b**). (**c**) displays residues of loop 210, linker and of the βSBD involved in the interaction necessary for the SBD docking.

Other contacts characterizing the NBD(ATP)-SBD(NR) equilibrium conformation are those between βSBD loops and loop210 (see Fig. 7 and Table 2). In particular, Lys214 is involved in stable interactions, both with the SBD (Asp481) and with the linker (Table 2): the same contact is not stable in the NBD(ATP)-SBD(free) complex (Fig. 6a) and is absent in the ADP complexes (Fig. 6b). These results confirm that loop 210 is an ATP-modulated key element for the linker docking and therefore for the allosteric communication between the two domains, as already suggested by our previous work^25^. Another interaction at the interface between SBD and NBD of the NBD(ATP)-SBD(NR) equilibrium structure is found between the conserved Asp481, in loop L_6,7_ and Thr173. Remarkably, mutation of Asp481 to Asn is known to cause weakening of the allosteric coupling between NBD and SBD induced by peptide^11^.

Moreover, we focused on the electrostatic couplings between Arg167 and Lys155 in the NBD and Asp387 and Asp393 in the linker region. In all cases, the NR-peptide reduces the average distances between Asp393 and the positively charged residues of NBD by ~1\AA (Supplementary Table S2). Interestingly, the mutation of Asp393 to Ala is experimentally found to interfere with the allosteric coupling (ATP-induced peptide dissociation), but not with the intrinsic ATPase rate^23^. The same holds for mutants of Arg167 and Lys155^23^, which suggests the specific role played by these residues in favouring the allosteric communication from SBD to NBD.

The equilibrium ensemble we observe in our trajectories of the NBD(ATP)-SBD(NR) complex shows some features of the allosterically active conformation proposed by Zhuravleva *et al.* in^11^, namely a docked linker and a limited contact interface between the two domains. In particular, we observe that in our MD data the presence of a substrate is necessary to form the parallel face-to-face arrangement; it significantly enhances the formation of a β-strand within the linker, which is stabilized by the βSBD trough several hydrogen bonds. In complexes bound to a substrate, the linker docking is also reflected in an increased coordination between lobe I and lobe II (Supplementary Fig. S5) compared to the apo form, which might explain the enhanced ATPase activity.

### Conformational modulation of βSBD

**Figure 8:**
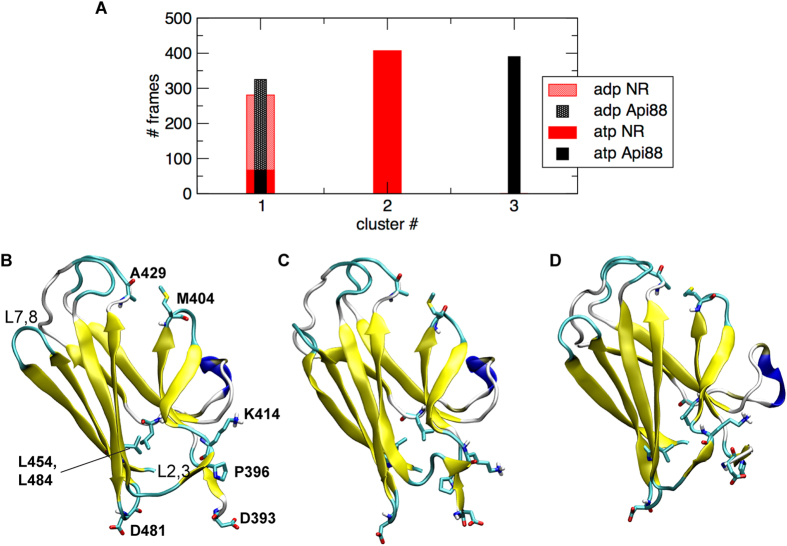
Substrate effect βSBD conformation. (**a**) histogram showing the population of the dominant three clusters broken down according to the nucleotide and ligand state. (**b**–**d**) cartoon representation of cluster centre conformations of first, second and third dominant cluster, respectively.

In this section we focus on the conformational evolution of the SBD domain in the different systems. Global conformational changes can either originate from local perturbations due to a bound peptide, or they can be triggered by the modulation of the interaction surface with the NBD, hence be allosterically driven by the NBD bound nucleotide. To get insight into this, we analysed, by cluster decomposition of the meta-trajectory (see Methods), the structural population of the βSBD (residues 393–503). We focused only on the βSBD, hence not considering the αSBD, which shows a strong flexibility, but does not undergo significant conformational changes in the different systems. The two most populated βSBD conformations (Fig. 8b,c) are characterized by different arrangements of the beta sandwich. The first cluster is populated by the undocked ADP state, both in the presence and in the absence of a substrate (Fig. 8a) and maintains the main features of the X-ray ADP bound DnaK (Fig. 8b). The second one is typical of the NBD(ATP)-SBD(NR) state (Fig. 8c), while the third one is exclusively populated by the inhibitor bound conformation NBD(ATP)-SBD(Api88) (Fig. 8d), in agreement with the previous linker/SBD decomposition analysis (Fig. 5). We observe that the combined effect of substrate and ATP-driven docking results in confining the SBD surface exposed to the NBD: in loop L_2,3_ the Cα-Cα distance from Pro396 and Lys414 decreases from 13 to 9 Å. As a consequence, loop L_6,7_ reduces its distance from the C-terminal end of the linker of 3 Å. In parallel, the hydrophobic core is slightly opened and the Cα-Cα distance between residues Leu454 and Leu484 increases by 2 Å. This analysis is in agreement with a recent study^29^ where the modulation of the hydrophobic core and of the SBD binding site upon peptide binding were observed by NMR chemical shift perturbation. Importantly, the different arrangements observed in the two NR-bound systems in the presence of ATP and ADP suggests that the docking of the SBD linker to the NBD interface determines the conformational change observed in the βSBD, rather than the NR-peptide itself. In contrast to the NR-bound system, the inhibitor bound βSBD shows, in the presence of ATP, some unique conformational features such as an increase of the tilting angle between the two beta sheets forming the sandwich. These results allow us to connect the modulation of loop L_2,3_ to the opening of the SBD binding site.

## Conclusions

In this paper we investigated the combined effect of multiple ligands bound to the NBD and to the SBD of DnaK, with the aim of dissecting the molecular determinants of the allosteric communication in both directions. By means of all-atom MD simulations we highlighted differences in the interdomain dynamics that result from a local modulation of the interconnecting linker, mainly at two hinges: Thr383 at the end of the NBD domain, and Asp393 at the connection to the SBD. Although on the simulated time scale we are not able to reproduce the whole conformational cycle of DnaK, significant global changes occur, which are related to the microscopic structural organization induced at the linker level by different bound ligands. On the basis of our results, we propose a model for the allosteric communication in DnaK. According to it, the NBD ligand and the SBD ligand act independently on the linker affecting each one of its ends through local conformational changes. Depending on the combination of the ligands, thanks to the flexibility of the linker and the local nature of the transitions, the global interdomain dynamics of NBD and SBD can be modulated and the conformational population easily shifted towards specific arrangements^28^. In particular, the effects of ATP and NR-peptide combine to generate a stable linker-docked complex, characterized by an increased coordination of lobe I and II forming the ATP binding site. The convergence of the dynamics, the persistence of the beta strand content of the docked linker, as well as the increased coordination in the ATP binding site, support the hypothesis that this structure could be a physiologically relevant intermediate on the pathway towards the ATPase active state of DnaK. Interestingly, inhibitor Api88 shows some similarity to the substrate peptide, as it increases the rigidity of the βSBD and consequently limits the conformational search of the linker when ATP is bound at the NBD. Consistent with this observation, Api88 has been observed to stimulate the ATPase rate of DnaK, albeit to a lower extent than the NR peptide. Our data suggest that the modulation of the relative dynamics of SBD and NBD, observed by MD simulations on the ns scale and quantified by the centre of mass analysis, could effectively reflect the efficacy of the allosteric communication between the domains, as well as the modulation of the ATPase function of Hsp70. This opens up the possibility of investigating and predicting the functional effect of introducing mutations or binding small molecules through an MD-based analysis of the inter-domain relative dynamics.

## Methods

### Protein structures

ADP and ATP complexes were obtained as described in the previous work^25^, stating from the X-ray structure of E.coli DnaK (PDB id: 2KHO)^20^. The NRLLLTG peptide was obtained from the X-ray structure of E.coli DnaK-SBD (PDB id: 1DKX)^26^. The apidaecin peptide (Api88) was retrieved from the X-ray structure of E.coli DnaK-SBD (PDB id: 4E81)^27^. In order to obtain the peptide complexes, the SBD of the ADP/ATP-apo forms were superposed to the X-ray structures co-crystallized with the peptides and peptide coordinates were transferred to the apo coordinates.

All complexes were solvated in a triclinic box of SPC water keeping a minimum distance of 1 nm between the solute and each face of the box. This results in about 40.000 water molecules included in the DnaK complexes. Total charge was neutralized with Na^+^ ions added to the simulation box at random positions.

### Molecular Dynamics simulations

MD simulations were performed with Gromacs 4.0 package^30^, employing the GROMOS96 (ff43a1) force field^31^. All complexes were energy relaxed with 1000 step of *steepest-descent* energy minimization. Two MD simulations for each complex were performed using the LINCS algorithm^32^ to constrain bond lengths and periodic boundary conditions were applied in all directions. Long-range electrostatic forces were treated using the Fast Particle-Mesh Ewald method (PME)^33^. Van der Waals forces were treated using a cut-off of 0.9 nm and the simulation time step was set to 2 fs. An initial velocity obtained according to a Maxwell distribution at 300 K was given to all the atoms. After 1 ns of all-atom restrained MD, production simulations were run in NVT environment employing V-rescale as temperature coupling algorithm, with reference temperature set at 300 K. All simulations reached equilibrium within a time interval between 2 and 20 ns.

### Molecular dynamics analyses

To evaluate the effects of peptides bound both on single residues and on protein domains and the intrinsic differences between apo and bound complexes, different analyses were carried out on the equilibrated trajectories. Several Gromacs tools were applied, such as *g_mindist* and *g_rms*, using default parameters.

#### Clustering

Several Cluster analyses were carried out using *g_cluster* module of Gromacs to evaluate the whole structure and the linker conformation using the gromos method^34^ on Cα atoms and different cutoffs. First, clustering of the concatenated replicas for each ligand state was carried out with a cut-off of 0.35 nm for the cluster analysis on the whole protein.

Clustering on the meta-trajectory obtained by concatenating the last 90 ns of each run (720 ns total) was performed focusing on different subsets: residues 373–393 for the linker cluster analysis, residues 393–433 for the βSBD analysis, both with a cutoff of 0.35 nm, and finally, on residues 393–503 for the βSBD clustering with a cutoff of 0.15 nm.

#### Hydrogen bond/contact analysis

The *g_hbond* module of Gromacs, applied on the single trajectories, and in-house developed scripts have been employed to highlight the interactions between the NBD and SBD domains and the linker. Residues establishing a hydrogen bond or a contact with a linker residue for at least 30% of the whole simulation time were collected.

#### Contact interface mapping

Lists of SBD residues within 6 Å of the NBD residues and of NBD residues within 6 Å of the SBD were obtained using the *g_mdmat* tool, which returns the averaged distance matrix over the whole trajectories of residues within a minimum distance of another set of residues.

#### Distance fluctuations matrix

For each MD trajectory, the distance fluctuations map *A* is calculated over the trajectory as:


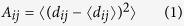


where *d*_*ij*_ is the (time-dependent) distance between the Cα atoms of amino acids *i* and *j* and the brackets indicate time-average over the simulation. Each matrix entry reports on fluctuation of the inter-residue distance in the corresponding residue pair. Lower distance fluctuation values correspond to a higher internal coordination between the residues (local rigidity). Matrix regions showing relatively low values identify protein sub-domains that move together (in coordination) while undergoing structural fluctuations^35^.

#### Centre of mass analysis

The trajectories were aligned fitting the NBD Cα atoms (residues 1–380) to the starting structure. The final 90 ns of two independent simulations were considered for each ligand state. The centre of mass of the SBD domain, defined by the interval of residues (393–603), was calculated for each trajectory snapshot, which allowed us to define a spatial distribution of positional coordinates.

## Additional Information

**How to cite this article**: Chiappori, F. *et al.* An atomistic view of Hsp70 allosteric crosstalk: from the nucleotide to the substrate binding domain and back. *Sci. Rep.* **6**, 23474; doi: 10.1038/srep23474 (2016).

## Supplementary Material

Supplementary Information

## Figures and Tables

**Figure 1:**
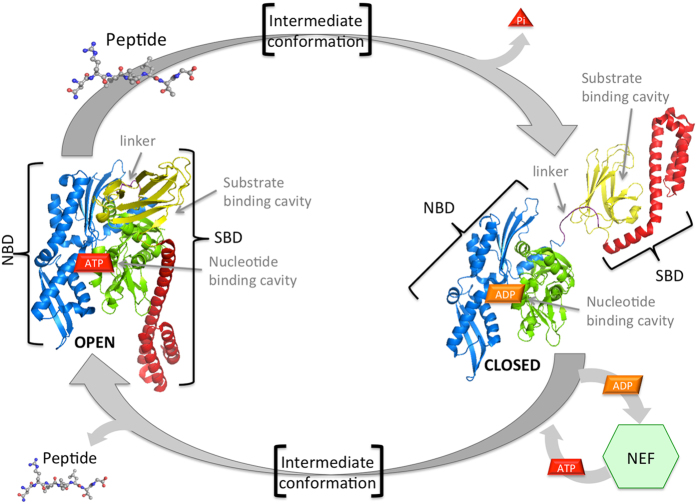
Hsp70 conformational cycle. Open (ATP-bound) (PDB id: 4B9Q) and Closed (ADP-bound) (PDB id: 2KHO) end-points X-ray structure are displayed. NBD lobe I is green, lobe II is blue, linker is violet, βSBD is represented in yellow, and αSBD helices in red. Peptide-substrate bound to the SBD binding cavity of the open conformation induces the ATP hydrolysis and a large structural rearrangement to the closed conformation. This, after nucleotide exchange mediated by Nucleotide Exchange Factors (NEF), undergoes to another conformational transition, with βSBD docked to the NBD and a reduced the affinity for the peptide-substrate.

**Table 1:** Contacts and hydrogen bonds involving the linker in the ADP-complexes.

Subdomain	Residues	ADP (1)	ADP (2)	ADP-Api88 (1)	ADP-Api88 (2)	ADP-NR (1)	ADP-NR (2)	2KHO
IA	ILE4						VAL389(c)	
	GLU137			THR395 (Hb)				
	LYS155		LEU391(c)		VAL394(c)	VAL394(c)		
	ARG159				VAL394(c)			
	VAL165	ASP393(c)	LEU391(c)		VAL394(c,Hb)		LEU392(c)	
			ASP393(Hb)					
	LYS166	LEU391(c)	LEU391(Hb)		LEU390(c)	ASP393(c)	VAL389(c)	
		LEU392(c)	LEU392(c,Hb)		LEU392(c)			
					ASP393(Hb)			
	ARG167	LEU391(c)	LEU390(c)	THR383(c)	LEU390(c)	THR383(c)	LEU390(c)	
						GLY384(c)		
	ILE168		VAL389(c)			ASP388(c)		
			LEU391(c)					
linker	GLN378			THR383(Hb)		THR383(Hb)		
	GLY379	THR383(Hb)		THR383(c,Hb)				
	GLY380	GLY384(Hb)			VAL386(Hb)		VAL386(c)	
	VAL381	VAL386(c,Hb)		VAL386(Hb)	VAL386(c,Hb)		VAL386(c)	
					LYS387(Hb)			
	LEU382	THR383(c)	THR383(c)	THR383(c)	LYS387(Hb)	THR383(c)	THR383(c)	
		ASP388(c,Hb)		ASP385(Hb)		VAL386(c)	ASP385(Hb)	
				VAL386(c)		LYS387(Hb)		
βSBD	LEU397	LEU390(c)	PRO396(c)	PRO396(c)	PRO396(c)	THR395(c)	PRO396(c)	
		PRO396(c)				PRO396(c)		
	LYS414		THR395(c)	ASP393(c)	PRO396(c)			
				PRO396(c)				
	ASN415	THR395(c,Hb)	THR395(c)	LEU391(c)	PRO396(c)	VAL394(c)	LEU390(c)	VAL394(Hb)
				LEU392(c)		PRO396(c)	ASP393(Hb)	THR395(Hb)
				ASP393(c)			PRO396(c)	
	THR416			LEU391(c)	LEU391(c)	THR395(Hb)	LEU390(c)	
					LEU392(Hb)			
					PRO396(c)			
	THR417				VAL389(c)			
					LEU391(c)			
	ILE418		LEU390(c)	VAL389(c)	VAL389(c)	VAL389(c)	LEU390(Hb)	
				LEU390(Hb)	LEU390(Hb)	THR395(c)	LEU391(c,Hb)	
							PRO396(c)	
	PRO419		LEU391(c)				ASP385(c)	
							ASP388(c)	
							VAL389(c)	
							LEU390(c)	
	THR420						GLY384(Hb)	
	HIS422						THR383(c)	
	ALA480		VAL389(c)	VAL389(Hb)				
			LEU390(Hb)					
	ASP481		ASP388(c,Hb)	LYS387(c,Hb)		VAL389(c)		LYS387(Hb)
			VAL389(Hb)			LEU390(c,Hb)		
						LEU391(Hb)		
	GLY482		VAL386(c)					
	LYS502		VAL386(c)					
αSBD	ALA503		VAL386(c)			LEU391(c)		
	SER504	VAL389(c)		ASP385(c)				
	SER505			ASP385(c)	THR395(Hb)		PRO396(c)	
	GLY506			THR395(Hb)				
	LEU507		GLY384(c)			LEU391(c)		
			VAL386(c)					
			PRO396(c)					
	GLU509	LEU390(c)				LEU391(c)		
		LEU391(c,Hb)				LEU392(c,Hb)		
	ILE512	PRO396(c)	VAL394(c)					
			PRO396(c)					
	GLN513	PRO396(c)		THR395(Hb)		LEU392(c)		
	VAL516			PRO396(c)		PRO396(c)		

**Table 2:** Contacts and hydrogen bonds involving the linker in the ATP-complexes.

Subdomain	Residues	ATP (1)	ATP (2)	ATP-Api88 (1)	ATP-Api88 (2)	ATP-NR (1)	ATP-NR (2)	4B9Q
IA	ILE4	GLY384(c)						
	**ASP148**					LEU392(c)	LEU391(c)	
	**ARG150**						LEU391(c)	
	**ARG151**					LEU390(c)		
						LEU391(c)		
	**GLN152**					LEU391(c)	LEU391(c)	
						LEU392(c)		
	LYS155					LEU391(c)		
	LYS166	GLY384(c)	ASP385(c)		LYS387(c)	THR383(c)	VAL394(c)	
		ASP385(c)	VAL386(c)		ASP388(c)			
	ARG167	VAL386(c)		THR383(c)	THR383(c)		THR383(c)	
							LEU390(c)	
							VAL394(c)	
	ILE168						LEU390(c)	
							LEU391(c)	
	ILE169						LEU390(c)	
	ASN170							ASP393(Hb)
	THR173							ASP393(Hb)
IIA	**VAL210**						VAL386(c)	
	**GLY212**						ASP385(c)	
	**GLU213**		THR383(Hb)	THR383(c)		GLY384(c)		
			LYS387(c,Hb)	GLY384(c)		ASP385(c)		
			ASP388(Hb)	ASP385(Hb)				
				VAL386(c,Hb)				
	**LYS214**					VAL386(c)	ASP388(Hb)	ASP388(Hb)
								LEU390(Hb)
	**THR215**						LYS387(c)	
	**PHE216**				THR383(c)		VAL386(c)	LEU390(Hb)
							LYS387(c)	LEU392(Hb)
	**VAL218**							LEU392(Hb)
linker	GLN378			THR383(Hb)	THR383(Hb)			
	GLY379		GLY384(c)			THR383(Hb)		
	GLY380				GLY384(Hb)	GLY384(Hb)		
	VAL381					VAL386(c,Hb)		
	LEU382	THR383(c)	THR383(c)	THR383(c)	THR383(c)	THR383(c)	THR383(c)	
						VAL386(c)	ASP385(c)	
							VAL386(c,Hb)	
βSBD	LEU397	PRO396(c)	THR395(c)	PRO396(c)	LEU390(c)	PRO396(c)	PRO396(c)	
			PRO396(c)		LEU392(c)			
					PRO396(c)			
	ILE412				LEU392(c)			
	LYS414			PRO396(c)				
	ASN415	PRO396(c)			ASP393(c)	VAL394(c)		THR395(Hb)
						THR395(c)		
	THR416	THR395(Hb)	THR395(Hb)		LEU391(c)			THR395(Hb)
	THR417				LEU391(c)			
	ILE418		THR395(c)	VAL389(c)	VAL389(c)		PRO396(c)	ASP393(Hb)
				LEU391(c,Hb)				
	PRO419						LEU392(c)	
	THR435					THR395(Hb)		
	ALA480			VAL389(Hb)			LEU391(c)	
							LEU392(Hb)	
	ASP481			LYS387(c,Hb)			LYS387(c,Hb)	
				ASP388(Hb)				
	GLY482						LYS387(c)	
							VAL389(c,Hb)	
	ILE483						ASP388(c)	
	LYS502				VAL386(c)		GLY384(c)	
							ASP388(c)	
αSBD	ALA503		VAL389(c)		VAL386(c)		PRO396(c)	
			LEU390(c)					
	SER504				ASP385(c)	LYS387(Hb)	VAL394(c)	
						ASP388(c)	THR395(c)	
							PRO396(c)	
	LEU507				VAL386(c)			
					ASP388(c,Hb)			
					LEU390(c)			
	ASN508				ASP388(c)	LYS387(c)		
	GLU509	LEU391(c)	LEU390(c,Hb)		LEU390(c)	LEU390(Hb)	THR395(Hb)	
		LEU392(c,Hb)	LEU391(Hb)			LEU391(c)		
						LEU392(c)		
	ASP510	LEU390(c)	LEU391(c)					
	ILE512	LEU392(c)		VAL394(c)	LEU390(c)	PRO396(c)		
				PRO396(c)	PRO396(c)			
	GLN513	VAL389(c)	LEU392(c,Hb)			VAL389(c)		
		LEU390(c)	PRO396(c)			PRO396(c)		
		LEU392(c)						
	VAL516		PRO396(c)		PRO396(c)	PRO396(c)		
