# Supplementary material for: An atomistic view of Hsp70 allosteric crosstalk: from the nucleotide to the substrate binding domain and back
Source: Sci Rep. 2016 Mar 30;6:23474. doi: 10.1038/srep23474 (PMC4812256; doi:10.1038/srep23474)
Supplement: Supplementary Information [file srep23474-s1.pdf]

An atomistic view of Hsp70 allosteric crosstalk: from the nucleotide to the substrate binding domain and back

Federica Chiappori[1], Ivan Merelli[1], Luciano Milanesi[1], Giorgio Colombo[2], Giulia Morra[2*]*

[1] Istituto di Tecnologie Biomediche – CNR, Segrate (Mi), 20090 Italy
[2] Istituto di Chimica del Riconoscimento Molecolare – CNR, Milano, 20131 Italy

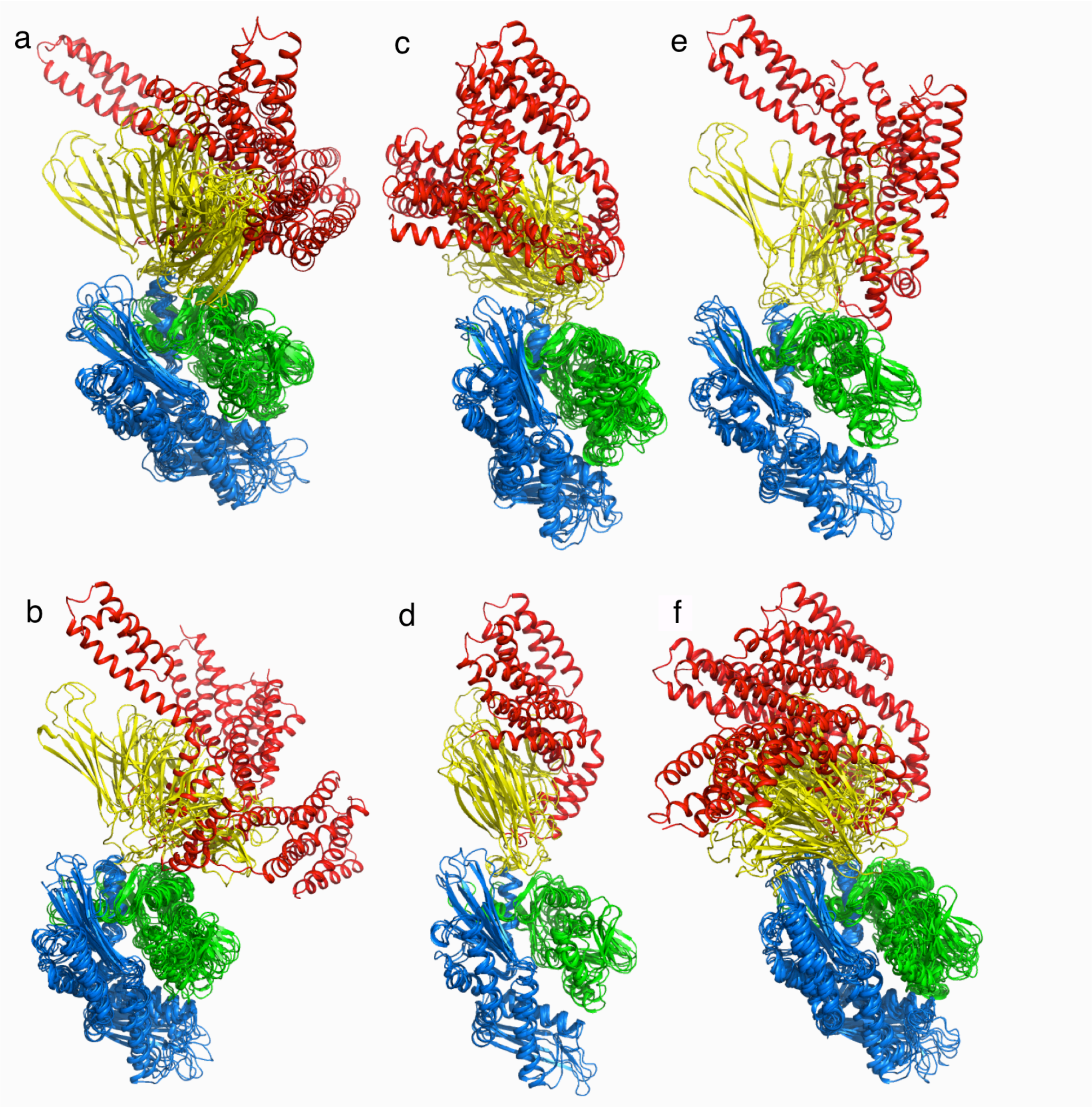

Figure S1 Representative conformations of all clusters. Cluster analysis was applied on concatenated trajectories for each complex. Conformations are superposed based on the NBD. a) NBD(ADP)-SBD(apo); b) NBD(ATP)-SBD(apo); c) NBD(ADP)-SBD(NR); d) NBD(ATP)-SBD(NR); e) NBD(ADP)-SBD(Api88); f) NBD(ATP)-SBD(Api88).

Table S1 Contact surface residues

NBD(ADP)-SBD(free)	NBD(ADP)-SBD(Api88)	NBD(ADP)-SBD(NR)	NBD(ATP)-SBD(free)	NBD(ATP)-SBD(Api88)	NBD(ATP)-SBD(NR)
135-138, 145-158, 163-167	134-138, 163-166	136, 154, 158, 163-167	113, 135-137, 150-166	150, 154, 166, 167	147-154, 158, 164-169
				212-215	213-216
377-383	377-383	382, 383	383	382, 383	380-383
388-396	388-397	388-394	388-391	388-390	388-395
414-421, 479-481	415, 418, 481	413-422, 480	442, 452-454, 483	480-483	480, 481
509	502, 504-510, 513, 517		500-514	502	502-509

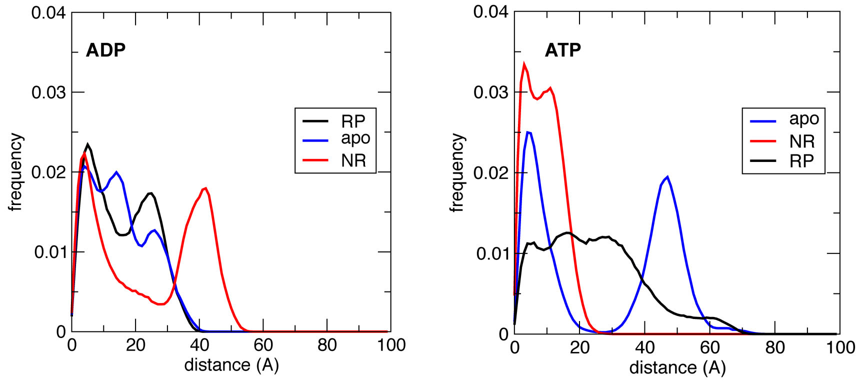

Figure S2 Pair distance distribution of SBD center of mass positions. Left, histogram showing the peaks in pair distances between different positions of the SBD center of mass in the presence of ADP and different SBD ligand states. Right, same with ATP in the NBD binding site.

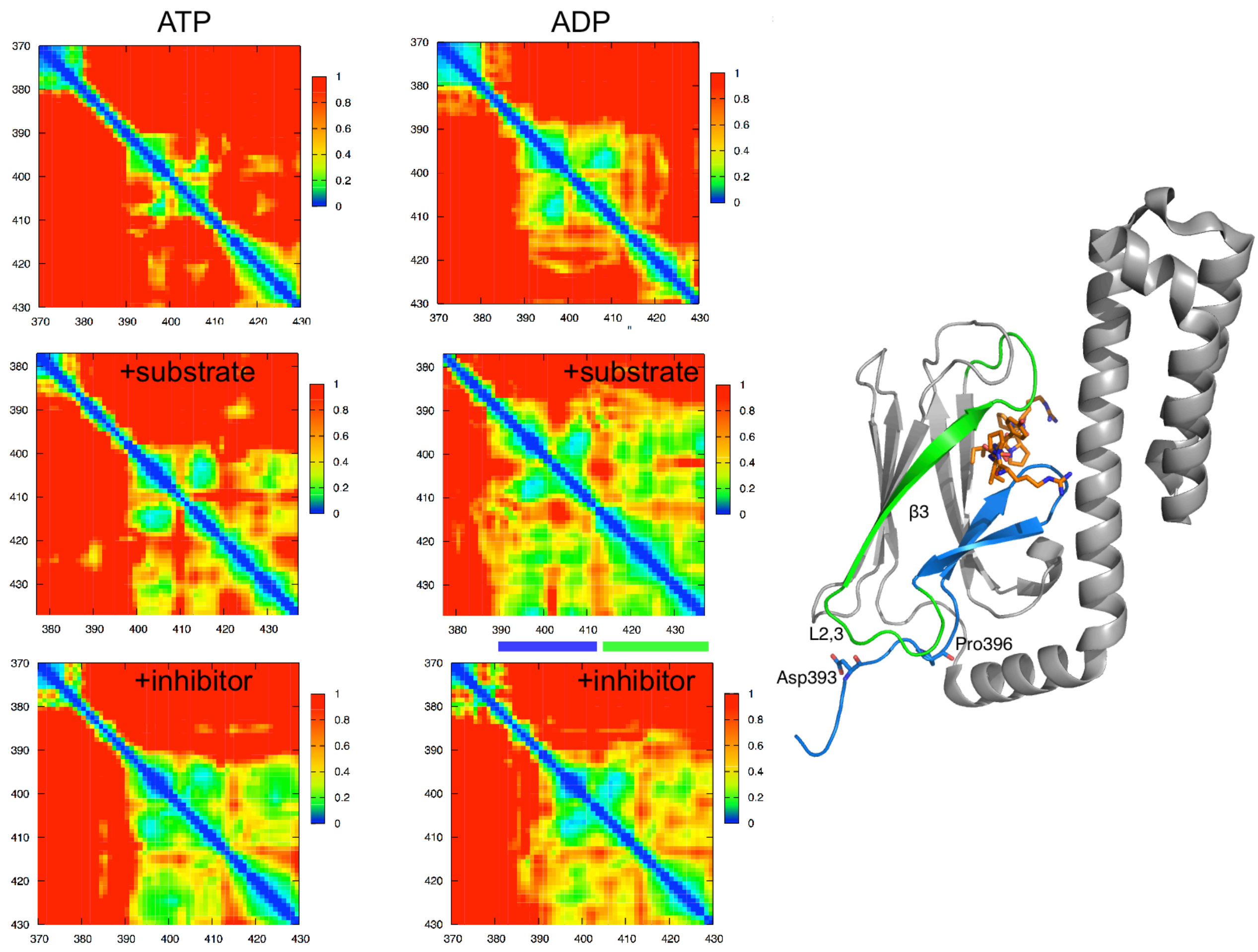

Figure S3 βSBD rigidity

Left panel. Distance fluctuation maps reporting on the pair distance fluctuations for residues 370-430 in the presence of ATP (left) and ADP (right) and in different ligand states: from top to bottom, SBD(free), SBD(NR), SBD(Api88). Right, structural representation of the SBD highlighting loop $L_{2,3}$ comprising residues 412-420.

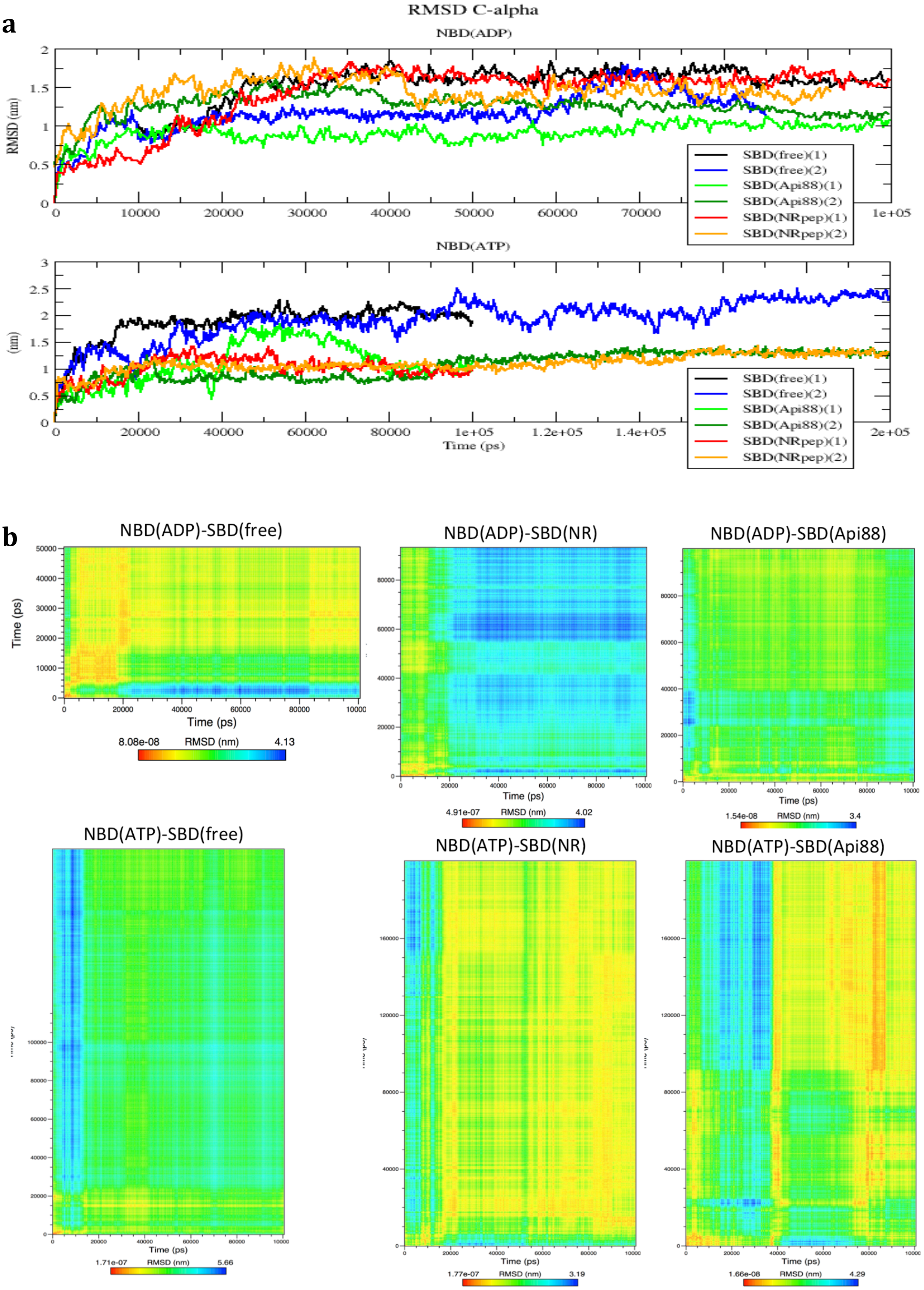

Figure S4 Simulation convergence. a) time dependent Cα RMSD. **b)** RMSD matrix of the Cα atoms of the two independent trajectories of each complex, obtained aligning the NBD, so that the RMSD mostly reports on the convergence and similarity of the interdomain arrangements

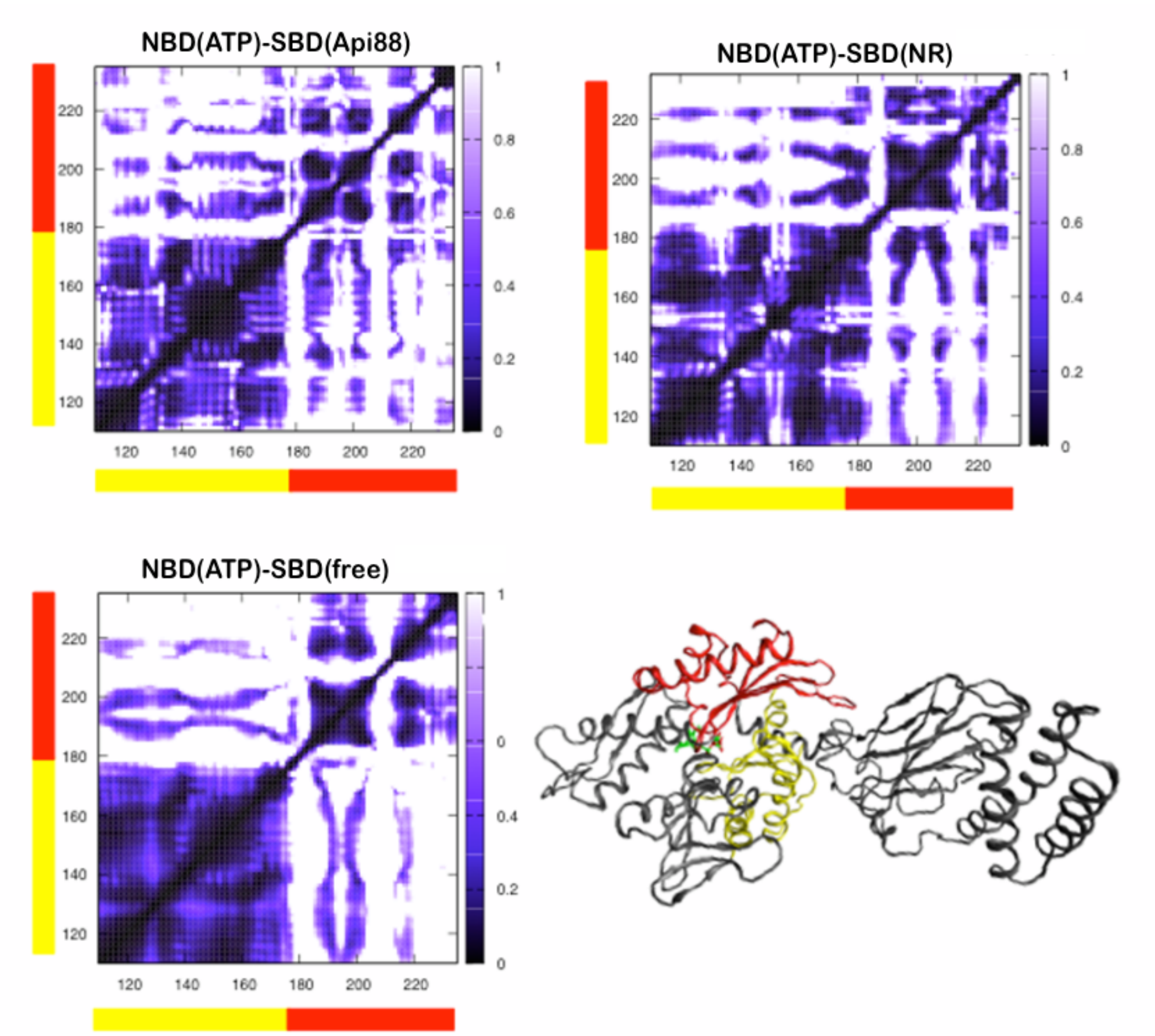

Figure S5 Lobe I – lobe II coordination. Matrix of the distance fluctuations calculated and averaged over the two independent trajectories, for each ATP complex. Bars distinguish lobe IB (yellow) and lobe IIB (red)

Table S2 Minimum distances (nm) reached during the simulation

mindist	Lys155-Asp393	Arg167-Asp393
ADP	0,79	1,28
ADP-Api88	0,68	0,62
ADP-NR	0,43	0,72
ATP	1,86	1,74
ATP-Api88	1,33	1,45
ATP-NR	0,60	0,95
